# Editorial: Mechanisms and innovations in combating intracellular infections

**DOI:** 10.3389/fmicb.2026.1892043

**Published:** 2026-06-18

**Authors:** Rajesh Mani, Manish Gupta, Nagendran Tharmalingam

**Affiliations:** 1 Department of Microbiology, Immunology and Molecular Genetics, University of Kentucky College of Medicine, Lexington, KY, United States; 2 Center for Tuberculosis Research, Department of Medicine, Johns Hopkins School of Medicine, Baltimore, MD, United States; 3 Department of Medicine, Houston Methodist Academic Institute and Research Institute, Houston, TX, United States; 4 Weill Cornell Medical College, New York, NY, United States

**Keywords:** antimicrobial resistance, autophagy, drug discovery, host-directed therapy, host–pathogen interactions, immune evasion, intracellular pathogens, translational innovation

## Introduction

Managing intracellular infections presents a remarkable challenge in modern medicine due largely to pathogens' ability to survive and replicate within protected host–cell niches. Immune evasion plays a significant role in the successful replication and persistence of intracellular pathogens, limiting drug penetration and immune recognition (Thakur et al., 2019). Classical intracellular pathogens such as *Mycobacterium tuberculosis* (Chandra et al., 2022), *Listeria monocytogenes* (Radoshevich and Cossart, 2018)*, Toxoplasma gondii* (Mani et al., 2022), and *Staphylococcus aureus* (Bose et al., 2020a,b; Tharmalingam et al., 2019) have evolved distinct survival adaptations. Despite occupying diverse niches, pathogens like *M. tuberculosis* in macrophages, *L. monocytogenes* in the blood–brain barrier and brain, and *T. gondii* in intracellular vacuoles share conserved strategies including phagosome maturation arrest and formation of protected intracellular compartments to aid their immune evasion and survival (Chandra et al., 2022).

Host defense is initiated by pattern recognition receptors which sense pathogen-associated molecular patterns, triggering cytokine and chemokine responses that shape downstream immunity (Akira et al., 2006; Mani and Suzuki, 2024). Innate cells, particularly macrophages and neutrophils, mediate early containment through phagocytosis, while autophagy serves as a critical antimicrobial mechanism frequently subverted by intracellular pathogens. Durable control requires effective T cell priming and effector function, comprised of Th1 CD4^+^ responses that enhance macrophage killing and CD8^+^ T cells that eliminate infected cells (Mani et al., 2022; Janeway et al., 2001). Antigen presentation by dendritic cells acts as a key bottleneck by determining which antigens are displayed to T cells (Mani et al., 2023). Many pathogens actively remodel this process, altering the timing and quality of T cell responses to enable persistence (Chandra et al., 2022).

Rising antimicrobial resistance and slow drug development intensify treatment challenges and underscore the need for non-antibiotic strategies (World Health Organization, 2025). Host-directed therapies (HDTs) and vaccine-based approaches are emerging as promising adjuncts to antimicrobial treatment (Mani et al., 2023; Afkhami et al., 2020; Mani et al., 2018). However, both the development and effectiveness of HDTs and vaccine-based approaches ultimately depend on a clear understanding of intracellular survival strategies and the immune pathways that they engage or subvert. This Research Topic focuses on intracellular survival mechanisms, immune sensing, and evasion, alongside innovative therapeutic strategies aimed at reducing antibiotic reliance. The collected studies identify vulnerable host–pathogen nodes and span mechanistic insights, translational studies, and enabling platforms. These contributions are synthesized across four themes: (1) host immune regulation, (2) intracellular adaptation, (3) pathogenesis, and (4) translational innovation.

## Theme 1. Host-directed immune regulation as a therapeutic lever

This Research Topic illustrates how host control nodes can act as both a defense mechanism and a pathogen target at the same time. The review on SUMOylation summarizes how SUMOylation shapes innate immune signaling (e.g., through pattern recognition receptors), inflammatory responses, and autophagy, and thereby, influences antibacterial defense mechanisms. Concurrently, this work highlights how bacterial pathogens hijack SUMO pathways by using effectors or by targeting SUMO enzymes and substrates, turning the host regulation into a virulence advantage (Xu et al.).

Building on host-pathway logic, the IL-22RA1 study demonstrates how intestinal epithelial IL-22RA1 signaling is a key gatekeeper that shapes mucosal containment of *Chlamydia muridarum*. Notably, when IL-22RA1 is absent in the intestine, *C*. *muridarum*, a strain lacking pGP3 plasmids, spreads further, reaching the large intestine with higher bacterial burden and shedding. Taken together, these studies position host immune regulation (via post-translational modifications and cytokine receptor pathways) as actionable switches for host-directed therapy developments (Tian et al.).

Tuberculosis (TB), primarily driven by *M. tuberculosis* persistence and the growing threat of drug resistance, remains the deadliest bacterial infection worldwide. A key review in this Research Topic highlights recent advances in three emerging TB treatment strategies: antimicrobial peptides, HDTs, and nanoparticle-based drug delivery systems. Beyond novel molecular targets, these innovative treatment strategies offer unique advantages. Additionally, the authors discuss recent advances in host–pathogen biology which have uncovered ferroptosis, a regulated form of iron-dependent cell death, as an important mechanism contributing to TB pathogenesis. Targeting these ferroptosis-associated mechanisms offers a promising avenue for therapeutic intervention (Zhang Y. et al.).

## Theme 2. Metabolism and pre-adaptation shape intracellular niches

The *Escherichia coli* Nissle 1917 (EcN) study frames intestinal inflammation as a three-way interaction from probiotic to pathogen to host, with metabolism as the key mediator. Mechanistically, arginine can blunt mitochondrial apoptosis signaling by modulating the RPS3-SRSF3 interaction, thereby stabilizing macrophage survival during infection. Notably, the authors introduce this as a new mechanism harnessed by EcN to inhibit macrophage apoptosis via arginine production during Salmonella challenge, ultimately suggesting a novel therapeutic angle for managing intestinal inflammation (Zhang L. et al.).

Complementing this, the *Mycobacterium avium* subsp. *hominissuis* (MAH) biofilm study argues that growth within a biofilm primes the bacteria for the macrophage microenvironment. Specifically, transcriptomics results show biofilm-grown MAH is transcriptionally closer to intracellular MAH than planktonic-grown bacteria, consistent with a pre-adapted state. These findings support the paradigm that preventing or disrupting biofilms may reduce downstream intracellular persistence and resilience (McManus et al.).

## Theme 3. Pathogenesis: tissue invasion and persistent reservoirs

Post-kala-azar dermal leishmaniasis (PKDL) remains an under-recognized complication of visceral leishmaniasis (VL) and poses a significant obstacle to leishmaniasis elimination efforts. The recent PKDL review from this Research Topic emphasizes how PKDL is not merely a dermatological sequel of VL but an outcome of complex interactions among host immune responses, parasite persistence, treatment protocols, and environmental factors. Importantly, the review highlights ongoing challenges in diagnosing and monitoring PKDL and underscores how future work combining genetic insights with better diagnostic tools and targeted treatments will be essential for controlling PKDL and maintaining progress toward global VL elimination (Mukherjee et al.).

*L. monocytogenes* is a zoonotic intracellular pathogen that infects the brain of ruminants and humans causing severe neurological infections. Despite its clinical significance, the mechanisms underlying this blood–brain barrier disruption and neuronal cell death remain poorly understood. The study by Hu et al. provides important insights into the neuropathogenesis of *L. monocytogenes*. Using a caprine infection model, the study critically demonstrates that infection triggers region-specific modulation of host cell death pathways, including apoptosis and autophagy, accompanied by dysregulation of tight junction proteins and activation of the E-cadherin/c-Met signaling axis.

## Theme 4. Clinical omics to screening platforms

On the clinical side, a *M. pneumoniae* bronchoalveolar lavage fluid proteomics study compared mild and severe pediatric disease to identify severity-associated signatures. Notably, the pathway enrichment results point to inflammatory programs, with IL-17 signaling emerging among enriched pathways, supporting the immune-driven severity biology. Together, this study provides a realistic translational direction for biomarker panels that may help risk-stratify or mechanistically subtype disease (Liang et al.).

In parallel, the Dd–Mm platform article addresses the pipeline gap by offering a scalable infection model for phenotypic screening. It uses a dual readout approach to measure pathogen growth and host cell viability in a high-throughput plate-reader format, enabling benchmarking and compound comparison. This platform further strengthens the discovery-to-mechanism workflows by helping prioritize candidates for deeper validation in higher-order models (Nitschke et al.).

Together, the studies in this Research Topic highlight the multifaceted nature of intracellular infections, spanning immune regulation, metabolic adaptation, tissue-specific pathogenesis, and translational innovation. A unifying concept is the identification of host–pathogen interface nodes that can be therapeutically targeted. By integrating mechanistic insights with emerging technologies and clinical data, these approaches provide a framework for developing next-generation interventions that complement traditional antimicrobials and address the growing challenge of antimicrobial resistance.
